# Whole genome sequencing and phylogenetic classification of Tunisian SARS-CoV-2 strains from patients of the Military Hospital in Tunis

**DOI:** 10.1007/s11262-020-01795-9

**Published:** 2020-10-09

**Authors:** Susann Handrick, Malena Bestehorn-Willmann, Simone Eckstein, Mathias C. Walter, Markus H. Antwerpen, Habiba Naija, Kilian Stoecker, Roman Wölfel, Mohamed Ben Moussa

**Affiliations:** 1 Bundeswehr Institute of Microbiology, Munich, Germany; 2 Department of Virology, Military Hospital of Instruction of Tunis, Tunis, Tunisia

**Keywords:** SARS-CoV-2, COVID-19, RT-qPCR, Sequencing, Tunisia, Morocco

## Abstract

In the present work, two complete genome sequences of SARS-CoV-2 were obtained from nasal swab samples of Tunisian SARS-CoV-2 PCR-positive patients using nanopore sequencing. The virus genomes of two of the patients examined, a Tunisian soldier returning from a mission in Morocco and a member of another Tunisian family, showed significant differences in analyses of the total genome and single nucleotide polymorphisms (SNPs). Phylogenetic relationships with known SARS-CoV-2 genomes in the African region, some European and Middle Eastern countries and initial epidemiological conclusions indicate that the introduction of SARS-CoV-2 into Tunisia from two independent sources was travel-related.

The current global spread of the newly emerged severe acute respiratory syndrome coronavirus 2 (SARS-CoV-2), the causative agent of coronavirus disease 2019 (COVID-19), has led to worldwide social and economic disruption. The virus was first described in December 2019 in Wuhan, China, and has since spread into a pandemic. As a result, healthcare systems in almost all countries of the world are facing unprecedented challenges. A few clinically approved drugs have been shown to exhibit anti-SARS-CoV-2 activity, but none have yet been proven to be sufficiently effective in treating COVID-19 patients, nor are vaccines available [1]. By August 2020, the World Health Organization (WHO) had reported more than 21.99 million infected individuals and 775,893 deaths worldwide. In the African Region, 966,352 confirmed cases have been reported [2], but many cases remain undetected, probably due to insufficient diagnostic capacity, limited contact tracing and the oftentimes unspecific symptoms of affected patients.

Diagnostic tests for SARS-CoV-2 have been carried out in Tunisia since early February 2020. On 2 March 2020, the first case of COVID-19, a Tunisian patient from Italy, was reported [3, 4]. Since then, the number of positively-tested SARS-CoV-2 patients has been increasing. By 18 August 2020, a total of 117,086 tests were performed and 2427 SARS-CoV-2 positive cases were reported by the Tunisian Ministry of Health. Of these positive cases, 60 patients died [5]. So far, only a few whole genome sequences from Tunisia are available online. However, these few data already indicate various independent, travel-related introductions of SARS-CoV-2 into the country [6]. Proper surveillance and monitoring of the SARS-CoV-2 epidemic in Tunisia and on the African continent, in order to guarantee faultless diagnostics, urgently requires further epidemiological and bioforensic data in addition to those currently available.

In the present work, two SARS-CoV-2 strains from Tunisian nationals were sequenced and phylogenetically compared with available SARS-CoV-2 genomes from Tunisia and other countries in Africa, Europe and the Middle East.

Four nasopharyngeal swab samples from Tunisian citizens were tested positive for SARS-CoV-2 RNA (Table 1) after viral RNA extraction with the QIAmp viral RNA Mini Kit (Qiagen, Hilden, Germany) using RT-qPCR according to the protocol of Corman et al. [7] at the Military Hospital in Tunis (MHT). The results of this investigation were confirmed at the Bundeswehr Institute of Microbiology in Munich (IMB) using an RT-qPCR protocol targeting the viral *N* gene [8]. An asymptomatic Tunisian soldier (31-year-old male, MHT_1) returning from a mission in Agadir, Morocco, tested positive immediately after his arrival at Tunis-Carthage Airport on 21 March 2020. In addition, three of five members of a Tunisian family from Ezzahra, 15 km south of Tunis, tested positive on 24 March 2020 (59-year-old mother, MHT_2; 62-year-old father, MHT_4) and 28 March 2020 (23-year-old daughter, MHT_3; Table 1). One of the patients, the father, showed symptoms such as fever, cough, arthralgia and headache one week before the first RT-qPCR test. The other two COVID-19 infections were without development of symptoms. All patients were tested negative for SARS-CoV-2 RNA in nasopharyngeal swabs after 14 days of home quarantine.

**Table 1:**
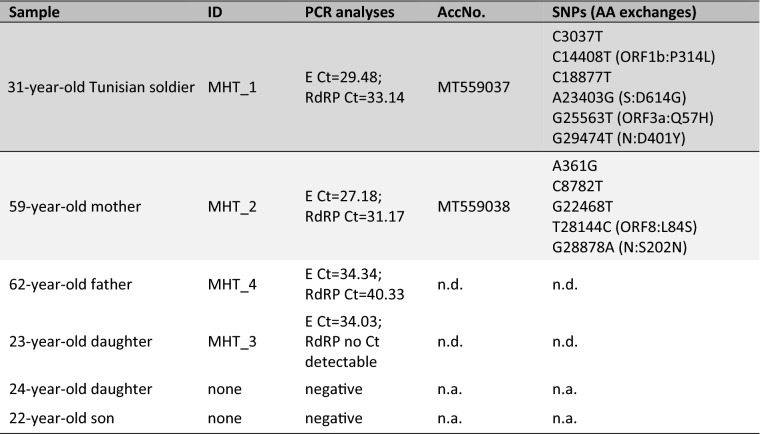
Patient information and sequencing results of MHT_1 and MHT_2

*n.d.* not done, *n.a.* not applicable

The four positively-tested RNA samples were further analysed at the IMB. For two of the four samples, having the highest virus loads (MHT_1/MHT_2; Table 1), whole genome sequencing of SARS-CoV-2 according to the nCoV-2019 sequencing protocol [9] on a GridION instrument was successful. Briefly, after passing quality control, demultiplexing, adapter trimming and consensus sequences generation based on the reference strain Wuhan-Hu-1 [10] using the ARTIC pipeline [11], closed genomes lacking only the outermost nucleotides of the 5′UTR or 3′UTR regions were obtained. Both genomes were annotated by and submitted to GenBank (accession MT559037 and MT559038).

SNP and phylogenetic analyses were performed using a local installation of the nextstrain.org pipeline [12]. Strains listed in GISAID belonging to the African subgroup (*N* = 1203 of 22 June 2020) and with available whole genome sequences were included in the initial analysis and further filtered phylogenetically to minimise redundancies and selected for relevance to possible travel and/or trade routes (*N* = 38).

In comparison to the reference strain Wuhan-Hu-1, MHT_1 shows six SNPs, four of which lead to amino acid changes in two different open reading frames (ORF) as well as the *S* and *N* genes respectively. Compared to Wuhan-Hu-1, MHT_2 contains five SNPs, two of which lead to amino acid changes in the ORF8 and the *N* gene. When comparing the two Tunisian SARS-CoV-2 sequences, no insertions or deletions and no mutual SNPs were found (Table 1).

Phylogenetic analyses showed that the genomes in our study are grouped in two different branches (Fig. 1). Sample MHT_1 belongs to clade 20A (Nextstrain.org nomenclature [13] matches Pangolin lineage B.1 [14]) with the characteristic mutation D614G (SNP A23403G) of the SARS-CoV-2 spike protein assumed to enhance viral infectivity [15]; sample MHT_2 is clustering within clade 19B (Pangolin lineage A). While genomes of clade 19 were dominated by Asian samples, especially during the first weeks of the incipient pandemic outbreak, clade 20A was the group rapidly spreading worldwide in February/March/April 2020. Observation of the genomes of both clones, which are present in North Africa, suggests multiple introductions at different points in time. When comparing whole genomes from Africa with a focus on the northern and central countries of this continent, the existence of geographically-associated genetic clusters can be observed.

**Fig. 1:**
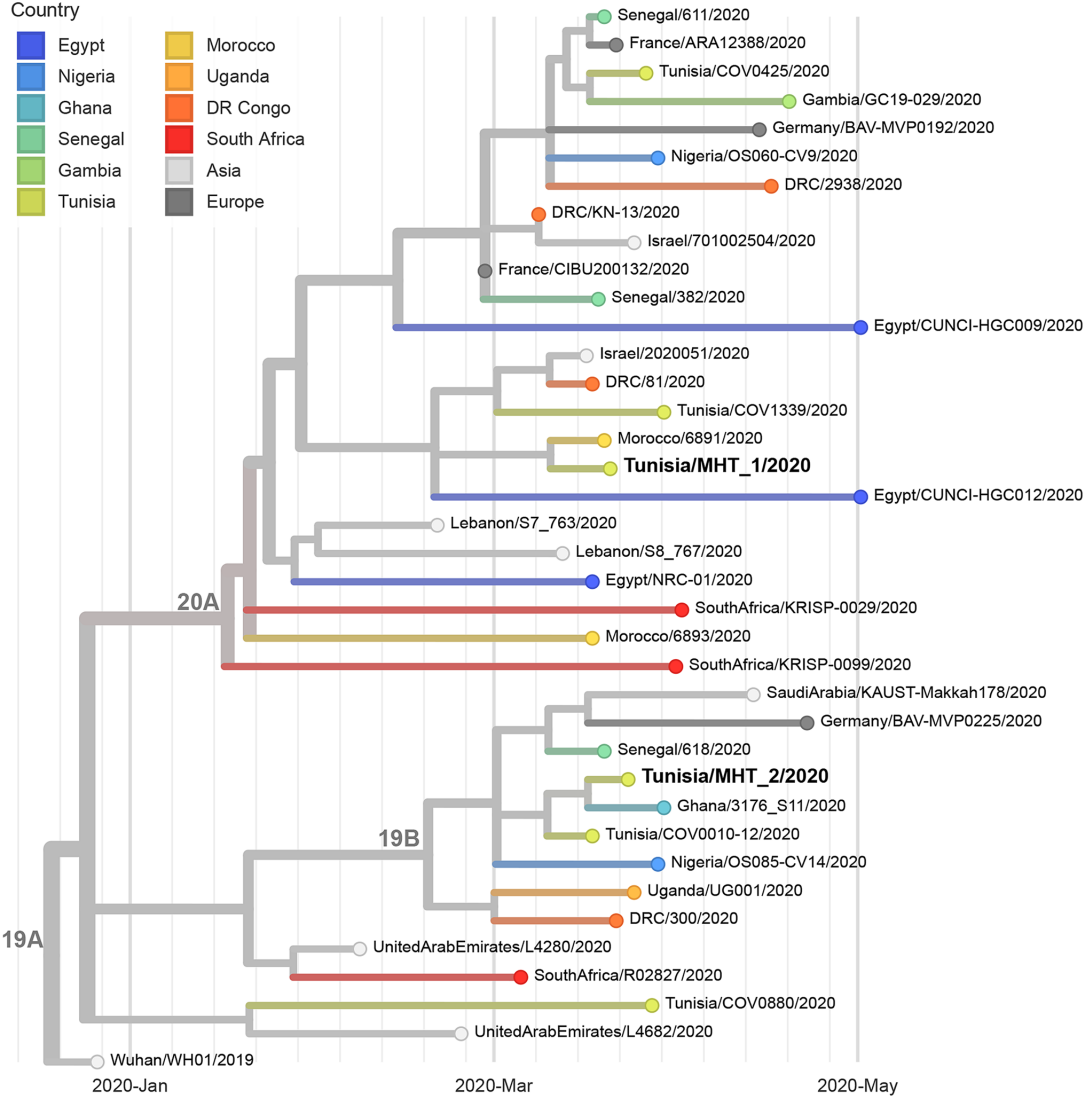
Phylogenetic tree showing the genomic epidemiology of a Tunisia-focused subsampling of 38 SARS-CoV-2 genomes sampled between Dec 2019 and May 2020. Unpublished data are included with the permission of the data generators and does not affect their right to publish. A complete list of sequence authors is available at nextstrain.org

Since the amount of genome sequences from the African continent is still small compared to European, Asian or American sequencing data, the interpretation of the results should be treated with caution. If more metadata are taken into account, possible epidemiological links become obvious and could explain genetic similarities. In pandemic situations, however, not all genetic similarities can be explained, even if data is available, e.g. the Bav-MVP0225/2020 sample from Germany. The situation is similar in the case of sample MHT_1, where the genetically closest neighbour is the isolate Morocco/6891/2020. This is conclusive, as this isolate originates from a Tunisian soldier who had returned from a stay in Morocco. The family isolate, on the other hand, is related to other virus strains, mainly from Central African countries such as Ghana, Uganda and the Democratic Republic of Congo (DRC). A direct link from Tunisia to these regions, e.g. on the basis of travel or trade, could not be established.

Therefore, correct and sufficient epidemiological data remain the greatest need for case tracing and successful infection control. In multidisciplinary approaches that take into account both epidemiological and genetic data, phylogeographic predictions can be verified on the basis of genetic analyses. While in epidemic or localised outbreaks with rapidly evolving pathogens, such as the Ebola virus, whole genomes can greatly assist in contact tracing, multiple introductions are difficult to monitor and track by phylogenetic analysis during prolonged outbreaks and pandemics. Medical and scientific cooperation is, therefore, one of the main triggers for combating pandemics like COVID-19.
